# Rhinoseptoplasty in a Blind Patient: A Case Report

**DOI:** 10.7759/cureus.29000

**Published:** 2022-09-10

**Authors:** Mohammed Elsayed, Roaa M Mandora, Bayan F Hafiz, Ahmad M Saad, Abdulrahman Kabli

**Affiliations:** 1 Otolaryngology Head and Neck Surgery, King Abdullah Medical City, Mecca, SAU; 2 Medicine and Surgery, College of Medicine, Umm Al-Qura University, Mecca, SAU; 3 Dentistry, Future University in Egypt, Cairo, EGY

**Keywords:** amygdala, self-perception, blind man, deviated nasal septum, nasal obstruction

## Abstract

Vision seems to be the first to recognize a potential threat, subconsciously recording and processing the image. Visual discrimination happens at a subcortical level after an environmental image is recorded in midbrain tissues. Aesthetics and beauty have been found to be decoded subconsciously in the amygdala, similar to a frightening threat. Therefore, blind patients can detect beauty by embodied primal senses other than vision. It could be processed without conscious thought, in the same way, that an immediate threat is. Here, we present a case of a 55-year-old male who has had bilateral blindness for 15 years and came to a rhinoplasty clinic seeking help for nasal obstruction and difficulty breathing due to an old history of trauma since adolescence, causing nasal deviation. He asked for both aesthetic and functional corrections. Rhinoseptoplasty was done successfully, significantly impacting the quality of life and psychosocial distress.

## Introduction

Blindsight refers to the remarkable residual visual abilities of patients suffering damage to the striate cortex [1]. Although vision seems to be the first sense used to recognize a potential threat, subconsciously recording and processing the image, visual discrimination happens at a subcortical level after an environmental image is recorded in midbrain tissues. The hypothalamus and autonomic nervous tract receive a message from the amygdala [2]. Aesthetics and beauty have been found to be decoded subconsciously in the amygdala, similar to a frightening threat [3]. This demonstrates that beauty is detected by embodied primal senses other than vision and could be processed without conscious thought in the same way as an immediate threat is [4]. Because of the nose’s central location on the face, the nasal shape is one factor that influences our personality development and body image [5]. A deviated septum is a disorder in which the nasal septum - the bone and cartilage that divides the nasal cavity in half - is considerably off-center or bent [6]. It can cause various symptoms such as nasal obstruction, trouble breathing via the nose, headaches, and facial pain [6]. If the patient continues to experience symptoms despite medical therapy, they can consider surgery to correct the deviated septum, called rhinoseptoplasty [7]. The surgical correction of the deviated nasal septum aims to enhance the nasal airway opening [8]. Because of its functional and cosmetic aspects, it has both physical and psychological impacts on patients [9]. In particular, it improves nasal obstruction and raises most patients’ quality of life [5]. However, we present the case of a 55-year-old man who has had bilateral blindness for 15 years that sought treatment for nasal obstruction and breathing difficulties at the rhinoplasty clinic at the King Abdullah Medical City in Mecca, Saudi Arabia.

## Case presentation

**Figure 1:**
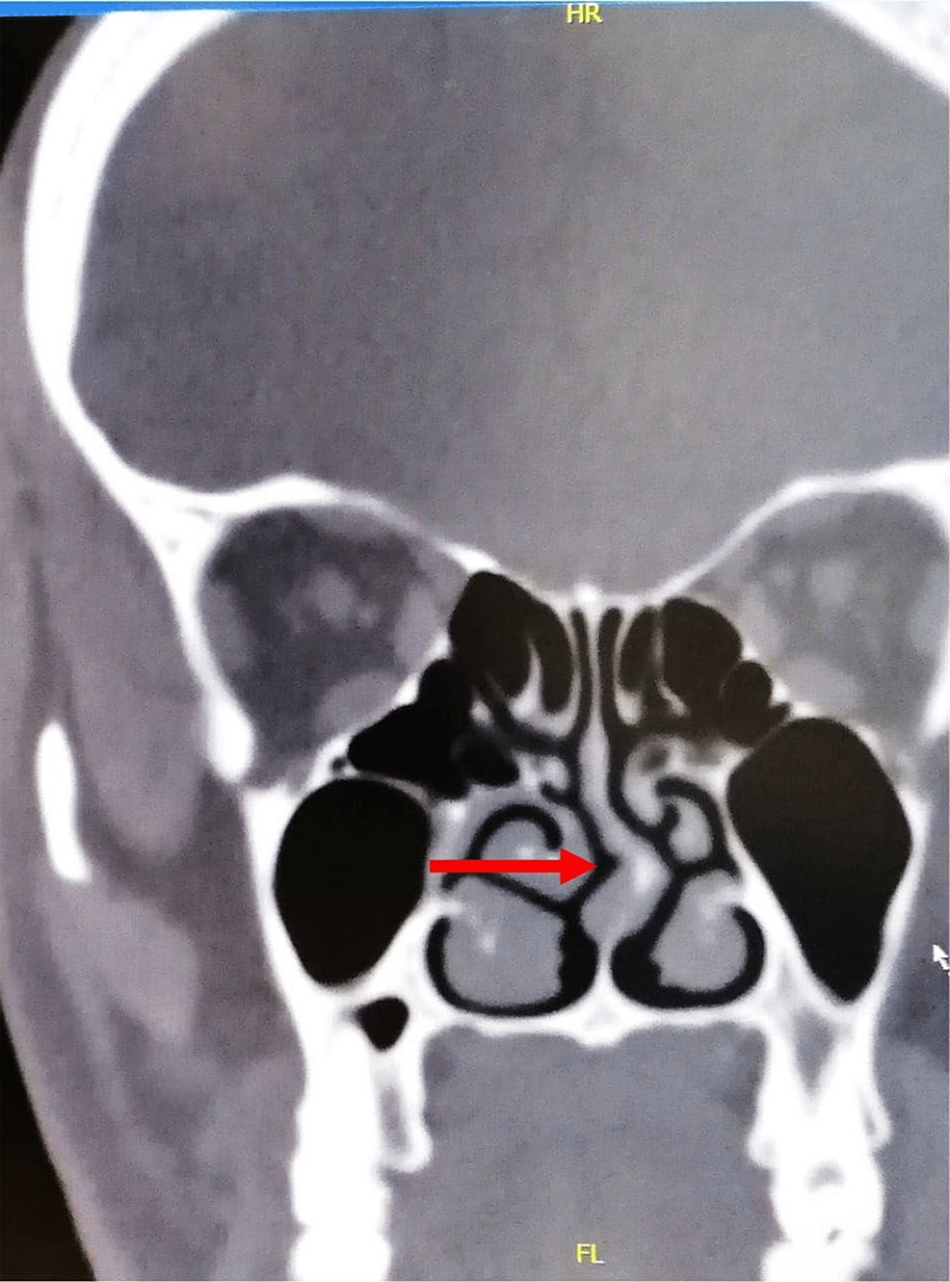
CT PNS showed extreme right septal deviation. CT PNS: CT scan of paranasal sinus cavities.

A 55-year-old man who has had bilateral blindness for 15 years presented at the rhinoplasty clinic at the King Abdullah Medical City in Mecca, Saudi Arabia, seeking help for nasal obstruction and breathing difficulties. The patient had no other complaints except for right-side nasal obstruction. He had no family history or past medical history and was in good physical and mental health. The patient’s complaint was due to an old history of trauma during adolescence, which caused the deviation of the axis of the nose to the right side, leading to a unilateral obstruction that caused his symptoms. This was also shown during the physical examination using flexible fiberoptic laryngoscopy. The CT of the paranasal sinus showed an extreme right septal deviation (Figure 1).

**Figure 2:**
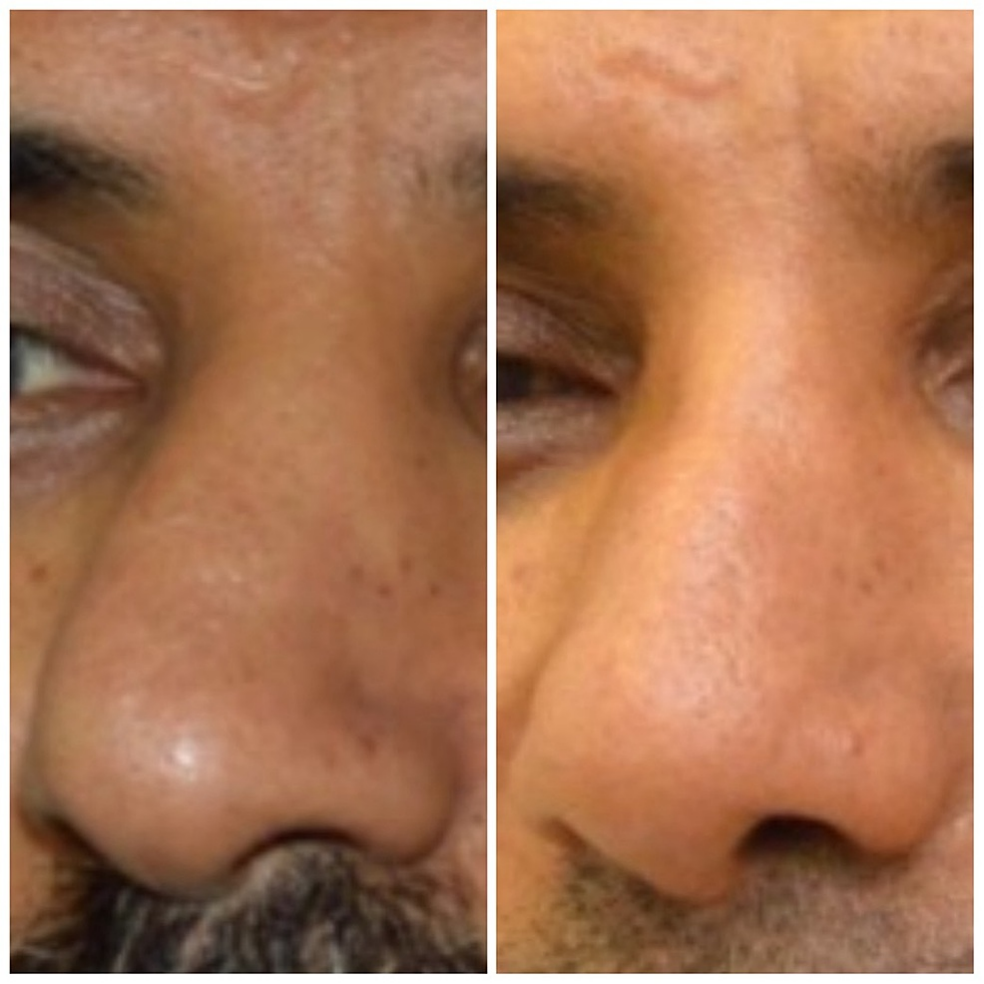
Before and after the rhinoseptoplasty.

Since the nasal trauma was before the patient lost his vision, he remembered his even nose structure before the trauma. Thus, he asked for both aesthetic and functional corrections. Rhinoseptoplasty was successful, and the patient was found to be in good health after the surgery. At the one-month follow-up post-surgery, the patient was satisfied. The previous symptoms of nasal obstruction and breathing difficulties had been resolved. Figure 2 shows the patient before and after the rhinoseptoplasty.

When the patient was asked to rate his level of satisfaction on a scale of 1 to 5 (1 = extremely dissatisfied and 5 = extremely satisfied), he rated a 1 before surgery and a 5 after surgery for nasal blockages, and a 3 before surgery and a 4 after surgery for nose shape fulfillment. His family and friends rated a 1 before surgery and a 3 on his nose shape.

## Discussion

Rhinoseptoplasty is a common otolaryngologic surgery. Indications for performing a rhinoseptoplasty include nasal obstruction, sleeplessness, and snoring. The most prevalent indication is a persistent nasal obstruction that does not respond to medical treatment [8]. Unfortunately, no objective criterion can be used to separate individuals who will react to medicinal treatment without requiring surgical intervention from those who will not respond to medical therapy and will need rhinoseptoplasty [9]. Additionally, the physiological impact and personal happiness may encourage rhinoseptoplasty if symptoms are present [10]. Thus, determining which individuals may benefit from rhinoseptoplasty clinically needs synthesizing several physical examination results rather than relying on one or two such findings.

Multiple studies have analyzed the impact of rhinoseptoplasty on a patient’s life [11,12]. These studies show that patients benefit from an increased quality of sleep and concentration and that symptoms such as a runny nose, nasal obstruction, and fatigue are resolved [11]. According to the literature, no cases of rhinoseptoplasty carried out in blind patients have been reported. We discussed the benefit and impact of this procedure for our patient with the Rhinoplasty Society of Europe, and they agreed to us to perform it.

In our case, the patient complained of nasal deviation with nasal obstruction and breathing difficulties. In addition, the patient was blind, which could affect the decision to perform surgery. However, we decided to proceed because the patient desired and consented to have the surgery. Furthermore, during the follow-up, he said he felt happy, comfortable, and more confident, enhancing his quality of life.

Does rhinoseptoplasty improve a patient’s quality of life? Yes, if it is performed in individuals with moderate-to-severe symptoms or if it improves the patient’s psychological condition [13]. However, the patient’s happiness and confidence play a significant part in deciding whether to perform the procedure to improve their quality of life.

## Conclusions

Several studies have shown that cortically blind patients can discriminate colors, simple shapes, and movements of objects even though they cannot see these stimuli. In this case, we managed the nasal septal deviation in a blind patient with rhinoseptoplasty, significantly improving his quality of life and lessening his psychosocial distress. Unfortunately, the literature on such surgeries for blind patients is lacking.
